# Comparison between **^18^**F-Fluorodeoxyglucose Positron Emission Tomography and Sentinel Lymph Node Biopsy for Regional Lymph Nodal Staging in Patients with Melanoma: A Review of the Literature

**DOI:** 10.1155/2011/912504

**Published:** 2011-12-22

**Authors:** Paoletta Mirk, Giorgio Treglia, Marco Salsano, Pietro Basile, Alessandro Giordano, Lorenzo Bonomo

**Affiliations:** ^1^Department of Bioimaging and Radiological Sciences, Institute of Radiology, Catholic University of the Sacred Heart, 00168 Rome, Italy; ^2^Department of Bioimaging and Radiological Sciences, Institute of Nuclear Medicine, Catholic University of the Sacred Heart, 00168 Rome, Italy

## Abstract

*Aim*. to compare ^18^F-Fluorodeoxyglucose positron emission tomography (FDG-PET) to sentinel lymph node biopsy (SLNB) for regional lymph nodal staging in patients with melanoma. *Methods*. We performed a literature review discussing original articles which compared FDG-PET to SLNB for regional lymph nodal staging in patients with melanoma. *Results and Conclusions*. There is consensus in the literature that FDG-PET cannot replace SLNB for regional lymph nodal staging in patients with melanoma.

## 1. Introduction

**^18^F** The usefulness of imaging studies in patients with melanoma generally depends on the stage of the tumour. In patients with early-stage disease, surgery is often curative with little role for comprehensive imaging in this patient population [1].

Regional lymph nodes are the most frequent site of metastatic disease. In many patients with primary cutaneous melanoma and clinically negative regional lymph nodes, surgical staging of the regional nodal basins at risk is performed using intraoperative lymphatic mapping and sentinel lymph node biopsy (SLNB). Regional nodal basins at risk (sentinel lymph nodes (SLN)) are typically identified by preoperative lymphoscintigraphy (LS) using intradermal injection of technetium-labelled sulphur colloid around the primary melanoma [2, 3].

Positron emission tomography with ^18^F-Fluorodeoxyglucose (FDG-PET) is a functional noninvasive imaging method extensively investigated in patients with melanoma [3].

We searched in the literature for relevant published articles which compared FDG-PET to SLNB for regional lymph nodal staging in patients with melanoma in order to assess if FDG-PET could substitute SLNB in this setting.

## 2. Comparison between FDG-PET and SLNB for Regional Lymph Nodal Staging in Patients with Melanoma: Literature Data

The first study on this topic was published in 1999 by Wagner et al. [4], who compared FDG-PET imaging of regional lymph node basins to SLNB, in patients with American Joint Committee on Cancer (AJCC) stages I, II, and III melanoma. Seventy patients with cutaneous melanoma with Breslow thickness greater than 1 mm (AJCC T2-4N0M0) or localized regional cutaneous recurrence (TxN2bM0) underwent whole-body FDG-PET followed by SLNB. Eighty-nine lymph node basins were evaluated by FDG-PET and SLNB. Eighteen patients (25.7%) had lymph nodal metastases at the time of FDG-PET imaging: 17 proved by SLNB (24.3%) and one by follow-up examination (1.4%). Median tumour volume in positive SLN was 4.3 mm^3^ (range: 0.07–523 mm^3^). Sensitivity of SLNB for detection of occult regional lymph node metastases was 94.4%, specificity was 100%, positive predictive value (PPV) was 100%, and negative predictive value (NPV) was 98.6%. Sensitivity of FDG-PET was 16.7%, specificity was 95.8%, PPV was 50%, and NPV was 81.9%. FDG-PET predicted one recurrence (14.3%) in a node basin missed by SLNB. The authors concluded that: (1) FDG-PET is an insensitive indicator of occult regional lymph node metastases in patients with melanoma because of the minute tumour volumes in this population; (2) FDG-PET has not a primary role for staging regional nodes in patients with AJCC stage I, II, or III melanoma [4].

Different results were reported by Klein et al. [5], who in 2000 investigated the use of whole-body FDG-PET in conjunction with LS for evaluating the status of SLNs in primary melanoma. A subgroup of 17 patients with primary cutaneous melanoma underwent LS, whole-body FDG-PET and SLN dissection. Out of 20 SLNs identified by LS in the 17 patients, 18 were negative on FDG-PET and 2 were positive; 19/20 PET findings were confirmed either by histopathology or by clinical followup (20 months). The accuracy of FDG-PET for the assessment of the status of the SLN was 94%. From this study, FDG-PET resulted in a reliable non-invasive alternative to surgery in the characterization of SLN [5].

In 2001, Acland et al. [6] compared the sensitivity of FDG-PET with SLNB in the detection of micrometastatic malignant melanoma. Fifty consecutive patients with primary melanoma (with thickness >1 mm or lymphatic invasion) underwent FDG-PET scanning followed by SLNB after preoperative LS. The SLN was identified in all patients; all patients with positive SLNB underwent therapeutic lymph node dissection. Fourteen patients had positive SLNB but in none of them FDG-PET identified metastatic disease in the SLN or draining basin. This study demonstrated the limitations of FDG-PET scanning in staging patients with primary melanoma. The authors concluded that SLNB has the disadvantage of being an invasive surgical procedure, but it is the only reliable method for identifying micrometastatic disease in the regional draining node with high sensitivity [6].

In 2002, Belhocine et al. [7] assessed the value of FDG-PET for detecting SLN metastases in 21 patients with early-stage melanoma (AJCC stage I or II) who also underwent lymphatic mapping and SLNB. Six of the 21 patients (28.5%) had an involved SLN. PET was positive in only one case with a SLN >1 cm. In the five other cases, the SLNs missed by PET were <1 cm with focal and/or partial involvements. One patient, free of regional nodal metastases in both SLN detection and PET imaging, had, however, a same-basin recurrence 3 months later. In another case, FDG-PET had one false positive result. Overall, the detection of subclinical nodal metastases by SLNB had a sensitivity of 86%. FDG-PET detected only 14% of SLN metastases. This study showed that SLNB remains the procedure of choice for detecting subclinical lymph node involvement from primary cutaneous melanoma. Owing to its limited spatial resolution, FDG-PET appears insufficiently sensitive to identify microscopic nodal metastases. As a practical consequence, FDG-PET imaging is not recommended as a first-line imaging strategy for staging regional lymph nodes in patients with AJCC stage I or II melanoma [7].

In 2003, Havenga et al. [8] reported the value of SLNB and FDG-PET in staging primary cutaneous melanoma. Fifty-five patients with primary cutaneous melanoma (with >1 mm Breslow thickness and no palpable regional lymph nodes) underwent FDG-PET and SLNB. SLNs were retrieved in 53 patients. Melanoma metastases were found in the SLN of 13 patients, but in only two of these 13 patients the lymph node metastases were detected by FDG-PET. Conversely, in five patients FDG uptake was recorded in a regional lymph node basin, but no tumour-positive SLN was found; no explanation for the positive FDG-PET result could be found in these five cases. The conclusions of this study were that: (1) FDG-PET should not be considered for staging regional lymph nodal disease in patients with primary cutaneous melanoma; (2) SLNB reveals regional metastases that are too small to be detected by FDG-PET [8].

In the same year, Longo et al. [9] compared FDG-PET imaging to SLNB for primary identification of lymph node involvement in patients with clinical staging I and II of cutaneous melanoma. Twenty-five patients with cutaneous melanoma (with a Breslow thickness equal or greater to 1 mm) underwent a preoperative FDG-PET to assess lymph node involvement. SLNB and FDG-PET showed a sensitivity of 100% and 22%, respectively, in the identification of lymph node metastases. The authors confirmed that FDG-PET is not a sensitive technique for the initial staging of patients with melanoma localized to the skin. The technique may nevertheless have a role for patients in whom SLNB is not indicated such as patients with a high surgical risk or those with prior wide local excisions that disrupt lymphatic drainage rendering SLNB less reliable [9].

In their study, Schäfer et al. [10] evaluated SLNB and FDG-PET in the staging of 51 melanoma patients (stages I and II according to the guidelines of the German Dermatological Society). Tumor thickness ranged from 1.0 mm to 6.0 mm. At least one SLN was excised in all patients; 80 SLNs were excised from 69 lymphatic drainage areas. Positive SLNs were detected in 6 patients (11.8%). Additional positive lymph nodes were not detected in any of these patients in the following lymph node dissection of the affected lymph node basin. Preoperative FDG-PET was performed in 40 patients and did not detect any of the micrometastases that were subsequently found by SLNB. During the followup of 7–40 months (mean 21.9 months), 3 patients experienced tumor progression; 2 of 3 had a positive SLN. These findings demonstrated that: (1) SLNB is recommendable in melanoma patients with primary tumors greater than 1 mm in thickness; (2) FDG-PET could not be expected to give additional information in the staging of stage I-II melanoma [10].

In 2004, Fink et al. [11] compared FDG-PET findings with histopathological results of SLNB in order to assess the value of FDG-PET in predicting regional lymph node involvement in 48 patients with primary stages I and II melanoma who underwent FDG-PET scans, preoperative LS, and SLNB. Of the 48 patients included in the study, eight (16.7%) had a positive SLNB. FDG-PET was positive in only one patient with a positive SLNB, yielding a sensitivity of 13%. All other positive SLNs could not be detected by FDG-PET imaging. This study confirmed that FDG-PET is not an adequate screening test for subclinical lymph node metastases in patients with stages I and II melanoma. The low sensitivity is probably due to the small size of metastatic deposits in SLN. Therefore, SLNB remains the technique of choice for evaluating the histological status of lymph node basins in patients with early-stage cutaneous melanoma [11].

In 2004, Hafner et al. [12] evaluated the sensitivity and specificity of SLNB and FDG-PET in the early detection of regional lymph node metastases in patients with melanoma. One hundred patients with melanoma and Breslow thickness over 1.0 mm were enrolled. SLNB was positive in 26 patients. FDG-PET detected two of 26 histologically tumour-positive SLNs (sensitivity 8%; specificity 100%). At 18-month followup, five of 26 (19%) patients with a positive SLN and four of 74 (5%) patients with a negative SLN had recurrent or progressive disease. Ninety percent of tumour-positive SLNs contained micrometastases with a diameter below the resolution of PET or US, which has been measured approximately at 4 mm. Therefore, radiological examinations such as US and PET cannot have a high sensitivity at baseline staging in patients with melanoma [12].

In 2005, Libberecht et al. [13] retrospectively evaluated the accuracy of SLNB and FDG-PET for early detection of lymph node metastases in 5 patients presenting with melanoma without clinical lymph node involvement and a Breslow thickness over 1 mm. In none of the patients the PET scan showed signs of lymph node involvement. However, two patients, both with a Breslow thickness of 1.4 mm, had micrometastases in the SLN which were missed by PET scanning. Therefore, the authors concluded that: (1) FDG-PET is of limited value in melanoma patients without palpable lymph nodes; (2) SLNB proved to be a useful tool and should be considered in the initial staging of melanoma without palpable lymph node or distant metastases [13].

In the same year, Vereecken et al. [14] evaluated the pertinence of a preoperative extensive staging procedure, including morphological and metabolic imaging and SLNB, in intermediate/high-risk melanoma patients. Forty-three patients with intermediate/poor prognosis primary melanoma benefited from complementary excision and SLNB after clinical and radiological staging (including FDG-PET scan). SLNB showed the presence of regional lymph node metastases in 10 patients, confirmed by the FDG-PET scan in four cases (sensitivity of FDG-PET: 40%). The authors confirmed that FDG-PET is not useful to detect lymph nodal micrometastasis and cannot replace SLNB in initial regional staging of patients with melanoma [14].

In their prospective study, Wagner et al. [15] determined the sensitivity and specificity of initial FDG-PET scan for detection of occult lymph node metastases in patients with early-stage cutaneous melanoma (inclusion criteria: tumours with >1 mm Breslow thickness, local disease recurrence, or solitary in-transit metastases without regional lymph nodal or distant metastases by standard clinical evaluation). FDG-PET findings in regional lymph nodes were compared with histology of SLNB specimens. SLNB and/or followup demonstrated regional lymph node metastases in 43 of 184 lymph node basins in 40 patients (27.8%). Compared with all clinical information, FDG-PET sensitivity for detection of regional lymph node metastases was 21% and specificity was 97%. FDG-PET did not impact the care of patients with early-stage melanoma already staged by standard techniques. Routine FDG-PET scanning was not recommended for the initial staging evaluation in this population because it resulted an insensitive indicator of occult regional lymph node metastases in patients with early-stage melanoma; it could not replace surgical techniques such as SLNB for staging of occult lymph node metastases in this population. The authors concluded that routine FDG-PET scan staging at the time of initial disease presentation does not have a significant clinical impact in patients with stages I and II melanoma who are candidates for lymph node staging with SLNB [15].

In 2006, Clark et al. [16] retrospectively reviewed 64 patients with T2 to T4 melanoma who underwent FDG-PET for detection of occult metastases at their institution. All patients underwent surgical excision of the primary lesion and SLN dissection. None of the patients had clinically suspected regional or distant metastases prior to FDG-PET. Nineteen of 64 patients had positive SLN, and only 2 (11%) were identified on FDG-PET. Overall, FDG-PET was not useful in predicting regional lymph node metastases neither changed the clinical management in any of the patients. This study suggested no utility for FDG-PET in the detection of occult metastases in patients at initial diagnosis of melanoma. The authors recommended the omission of FDG-PET imaging from preoperative evaluations for patients with melanoma; although they continued to suggest FDG-PET evaluation for selected patients with signs and symptoms of metastatic melanoma, they reported no role for routine FDG-PET imaging for asymptomatic patients who will be evaluated with SLN mapping. Further, it remains clear that negative FDG-PET imaging cannot be considered a viable substitute for SLNB [16].

In 2007, Kell et al. [17] examined the role of FDG-PET/CT in patients undergoing SLNB for early-stage melanoma. Patients presenting with primary melanoma without evidence of either locoregional or systemic metastasis were considered candidates for SLNB. Selected patients underwent preoperative FDG-PET/CT followed by definitive surgical therapy including SLNB with regional lymphadenectomy, where indicated. During a 12-month period, 83 patients underwent SLNB for melanoma, of which 37 (45%) had preoperative PET/CT. Thirteen (15.6%) patients were found to have lymphatic metastasis at SLNB; among nine of these patients who underwent FDG-PET/CT, only two PET scans were suggestive of lymphatic metastasis (PPV: 24%, NPV: 76%). FDG-PET/CT revealed no unheralded metastatic disease but identified a second occult malignancy in 4 (10.8%) patients undergoing therapy for melanoma. These findings demonstrated that FDG-PET/CT is not a useful adjuvant to lymphatic staging in patients with primary melanoma without signs of lymphatic metastasis. SLNB is a more sensitive tool in the detection of lymphatic metastasis. However, the results of this study support the concept that FDG-PET/CT may have some use in screening for unheralded occult primary malignancy, although the financial and quality of life implications of this have not been studied [17].

In the same year, Maubec et al. [18] prospectively determined the value of FDG-PET scanning in the detection of regional metastasis in 25 patients referred for the treatment of a primary melanoma thicker than 4 mm. SLNB was proposed for all the patients without a palpable regional lymph node. FDG-PET identified 0/2 primary melanomas, 1/4 residual primary melanomas after limited excision, 0/6 lymph node basins with micrometastasis, 4/4 lymph node basins with enlarged palpable lymph nodes, and 0 distant metastasis. The sensitivity and specificity of FDG-PET for microscopic lymph node disease in basins were, respectively, 0 and 92%. In the authors' experience, it is not useful to include FDG-PET in the initial workup of patients with primary melanoma, even in patients with thick primary melanomas (>4 mm), and SLNB remains the technique of choice for the most accurate initial staging [18].

In their study, Yancovitz et al. [19] examined the impact of preoperative radiologic imaging (including FDG-PET/CT), focusing on T1b to T3b melanoma patients without clinical evidence of nodal or distant disease. One hundred forty-two patients underwent SLNB, of whom 22 (15.5%) had positive results. Six of 22 patients with positive SLNB had preoperative FDG-PET/CT, but only one PET/CT study predicted the positive nodal basin. The study suggested that asymptomatic patients with ≥4 mm thick melanomas do not warrant extensive radiologic work-up: imaging at the time of initial diagnosis of T1b-T3b, clinically N0, M0 melanoma was of low yield with a high false-positive rate and did not lead to upstaging or changing the initial surgical management. Therefore FDG-PET/CT imaging of asymptomatic melanoma patients at the time of diagnosis may not be warranted [19].

In 2008, Singh et al. [20] evaluated the role of preoperative FDG-PET/CT, LS, and SLNB in 52 patients with AJCC stage I or II melanoma. None of the patients had clinical or radiological evidence of regional lymph node metastatic disease. At least one SLN was identified in all patients. Fourteen out of the 52 patients (27%) had at least one involved SLN. FDG-PET/CT was true positive in two patients with a SLN greater than 1 cm and false positive in two other patients. In this study, FDG-PET/CT imaging demonstrated very low sensitivity (14.3%) and PPV (50%) for localizing the subclinical nodal metastases. The specificity, NPV, and diagnostic accuracy were 94.7, 75, and 73%, respectively. Preoperative FDG-PET/CT imaging was not able to substitute SLNB in patients with stage I or II melanoma [20].

Recently, Klode et al. [21] sought to clarify the role of SLNB in the evaluation of the initial stages of 61 patients with primary melanoma (AJCC stages I and II; Breslow thickness >1 mm) by comparing it directly with FDG-PET/CT evaluation. Metastatic SLNs were found in 14 patients (23%); 17 metastatic lymph nodes were detected overall, only one of which was identified preoperatively using FDG-PET/CT. Thus, PET/CT showed a sensitivity of 5.9% and an NPV of 78%. The results of this study showed that initial-stage FDG-PET/CT in patients with AJCC stage I or II melanoma does not offer any advantage over SLNB with respect to the detection of local and regional metastases, because lymph nodal micrometastases cannot reliably be detected. SLNB, on the other hand, is a more sensitive procedure to diagnose lymph nodal metastases of melanoma and continues to be the criterion standard for the classification and stratification for adjuvant therapy of patients with AJCC stage I or II melanoma [21].

## 3. Remarks and Conclusions

Overall, with a few exceptions [5], the results reported by the studies in the literature are remarkably homogeneous in pointing out the limitations of FDG-PET/CT for regional lymph nodal staging in patients with cutaneous melanoma. Indeed in patients with early-stage disease (AJCC stage I or II, without regional lymph nodal or distant metastases), the sensitivity of FDG-PET/CT is unacceptably low, with most reported values ranging from 0% to 22% [4, 7, 9, 11, 12, 15, 16, 18, 20, 21]. Slightly higher- and yet too low-sensitivity values (40%) were found only for intermediate/high-risk melanoma patients [14], in whom metastatic adenopathy is more likely.

In early-stage melanoma SLNB, typically performed following intraoperative lymphatic mapping is now the reference standard for regional lymph nodal staging when there is no clinical evidence of regional nodal metastasis. The procedure is highly accurate for detecting occult lymph nodal metastases, with a false-negative rate ≤5% for experienced examiners. When there is clinical suspicion of metastatic adenopathy, ultrasound of the nodal basin with fine-needle aspiration (FNA) or biopsy under ultrasound guidance may otherwise be performed; if cytology is positive for malignancy, the patient is sent directly to radical lymph node dissection and SLNB is thus not necessary.

In the event of a negative sentinel lymph node biopsy, the patient is considered to have clinical stage I or II disease and no imaging is indicated: indeed, all imaging methods, including US, CT, and FDG-PET or PET/CT, have limited utility for the early stages of melanoma, with very low diagnostic yield and a burden of false-positive results leading to unnecessary workup.

FDG-PET/CT has an extremely limited role, if any, in microscopic nodal disease detection in patients with clinical stages I and II disease, since normal-size lymph nodes may contain micrometastases below the sensitivity threshold of PET/CT. In this patient population, SLNB is much more sensitive than PET/CT in discovering small lymph node metastases. In conclusion, FDG-PET and FDG-PET/CT are unsuitable for the evaluation of early regional lymphatic tumour dissemination in patients with AJCC stage I or II melanoma, and they should be limited to staging patients with more advanced (AJCC stage III or IV) melanoma.
